# Use of Cap Analysis Gene Expression to detect human papillomavirus promoter activity patterns at different disease stages

**DOI:** 10.1038/s41598-020-75133-2

**Published:** 2020-10-22

**Authors:** Ayumi Taguchi, Kazunori Nagasaka, Charles Plessy, Hiroe Nakamura, Yoshiko Kawata, Sachi Kato, Kosuke Hashimoto, Takeshi Nagamatsu, Katsutoshi Oda, Iwao Kukimoto, Kei Kawana, Piero Carninci, Yutaka Osuga, Tomoyuki Fujii

**Affiliations:** 1 Department of Obstetrics and Gynecology, Graduate School of Medicine, The University of Tokyo, Tokyo, Japan; 2 Department of Obstetrics and Gynecology, Teikyo University School of Medicine, Tokyo, Japan; 3 Okinawa Institute of Science and Technology Graduate University, Okinawa, Japan; 4 Division of Genomic Medicine, RIKEN Center for Integrative Medical Science, Yokohama, Japan; 5 Pathogen Genomic Center, National Institute of Infectious Diseases, Tokyo, Japan; 6 Department of Obstetrics and Gynecology, Nihon University School of Medicine, Tokyo, Japan

**Keywords:** Infectious-disease diagnostics, Virology, Cervical cancer, Biotechnology, Cancer, Computational biology and bioinformatics, Microbiology, Oncology

## Abstract

Transcription of human papillomavirus (HPV) genes proceeds unidirectionally from multiple promoters. Direct profiling of transcription start sites (TSSs) by Cap Analysis Gene Expression (CAGE) is a powerful strategy for examining individual HPV promoter activity. The objective of this study was to evaluate alterations of viral promoter activity during infection using CAGE technology. We used CAGE-based sequencing of 46 primary cervical samples, and quantitatively evaluated TSS patterns in the HPV transcriptome at a single-nucleotide resolution. TSS patterns were classified into two types: early promoter-dominant type (Type A) and late promoter-dominant type (Type B). The Type B pattern was more frequently found in CIN1 and CIN2 lesions than in CIN3 and cancer samples. We detected transcriptomes from multiple HPV types in five samples. Interestingly, in each sample, the TSS patterns of both HPV types were the same. The viral gene expression pattern was determined by the differentiation status of the epithelial cells, regardless of HPV type. We performed unbiased analyses of TSSs across the HPV genome in clinical samples. Visualising TSS pattern dynamics, including TSS shifts, provides new insights into how HPV infection status relates to disease state.

## Introduction

Uterine cervical cancer is the second most commonly diagnosed cancer, and the third leading cause of mortality among women in developed countries^1^. Persistent infection with high-risk human papillomaviruses (HR-HPVs) is the main cause of cancer development^2–4^. During the last two decades, HPV-induced carcinogenesis has been extensively studied. The HPV-derived E6 and E7 oncoproteins inactivate the p53 and pRb tumour suppressor proteins, respectively, which results in resistance to apoptosis and promotion of cell proliferation. Continuous high expression of E6 and E7 is the most important factor in cervical cancer progression^5,6^.

HPV transcription is unidirectional and generates numerous viral transcripts via differential RNA splicing. At least 13 transcripts from eight HPV genes were identified in HPV-16–infected W12E cells^7^. These transcripts overlap, complicating the evaluation of expression levels of each transcript. There are two major promoters in the HPV genome: the early promoter, located in the long control region (LCR); and the late promoter, located in the E7 gene downstream of the early promoter. The early promoter controls the expression of E6 and E7, while the late promoter regulates the expression of E1, E2, E4, and E5 (which are important for cell differentiation and viral replication), as well as the expression of L1 and L2 capsid protein genes^8^. The activity of these promoters is regulated by cellular transcription factors, or by the epigenetic alteration of the viral genome.

A transcriptome profiling method known as Cap Analysis Gene Expression (CAGE)^9^ can be used to determine the 5′-terminal sequence of RNA, allowing for promoter detection and quantitative measurement of promoter activity. The two main CAGE protocols currently used are no-amplification non-tagging (nAnTi)-CAGE^10^, which does not involve PCR amplification, and nanoCAGE^11^, which is designed to process samples that yield nanograms of RNA. nanoCAGE is based on PCR amplification, with the PCR bias removed through the use of unique molecular identifiers^12^. We previously reported that nAnTi-CAGE may be used to identify precise transcription start sites (TSSs) in the HPV genome, and have used this technology to quantify the activity of multiple promoters in three cell lines and one patient sample^13^. Direct evaluation of TSSs may represent a novel diagnostic strategy to assess HPV infection status and disease progression.

HPV genes are differentially expressed in parallel with the differentiation programme of the cervical epithelium. At the initial stage of HPV infection, the copy number of the viral genome in cells in the basal layer of the cervical epithelium is very low. Viral DNA replication proceeds along with epithelial differentiation^14,15^. In the upper epithelial layers, the viral late genes L1 and L2 are expressed to allow viral capsid assembly, packaging, and shedding from the superficial layer of the epithelium. As the viral late gene expression is promoted, E2 suppresses the activity of the early promoter by binding to the E2 binding sites (E2BS) of the LCR^16–18^. Thus, in the late stages of epithelial differentiation, HPV early promoter activity is relatively suppressed. As the severity of cervical intraepithelial neoplasia (CIN) increases, sustained high expression of E6 and E7 is driven by the early promoter, and, conversely, L1 gene expression is suppressed^19^. Several methods have been devised to evaluate the expression of late genes, such as L1 or E4, as biomarkers for CIN progression^19–25^. Precise evaluation of the late gene expression patterns could support their use as novel biomarkers for cervical cancer progression.

In this context, we propose that a quantitative assessment of promoter activity, by evaluating TSS activity, would allow for classification of HPV status, as well as CIN severity. In the present study, we developed a novel approach for the evaluation of differences of viral promoter activity at the single-nucleotide level using CAGE technology in clinical HPV samples.

## Results

### HPV TSS patterns of cervical lesions

**Figure 1:**
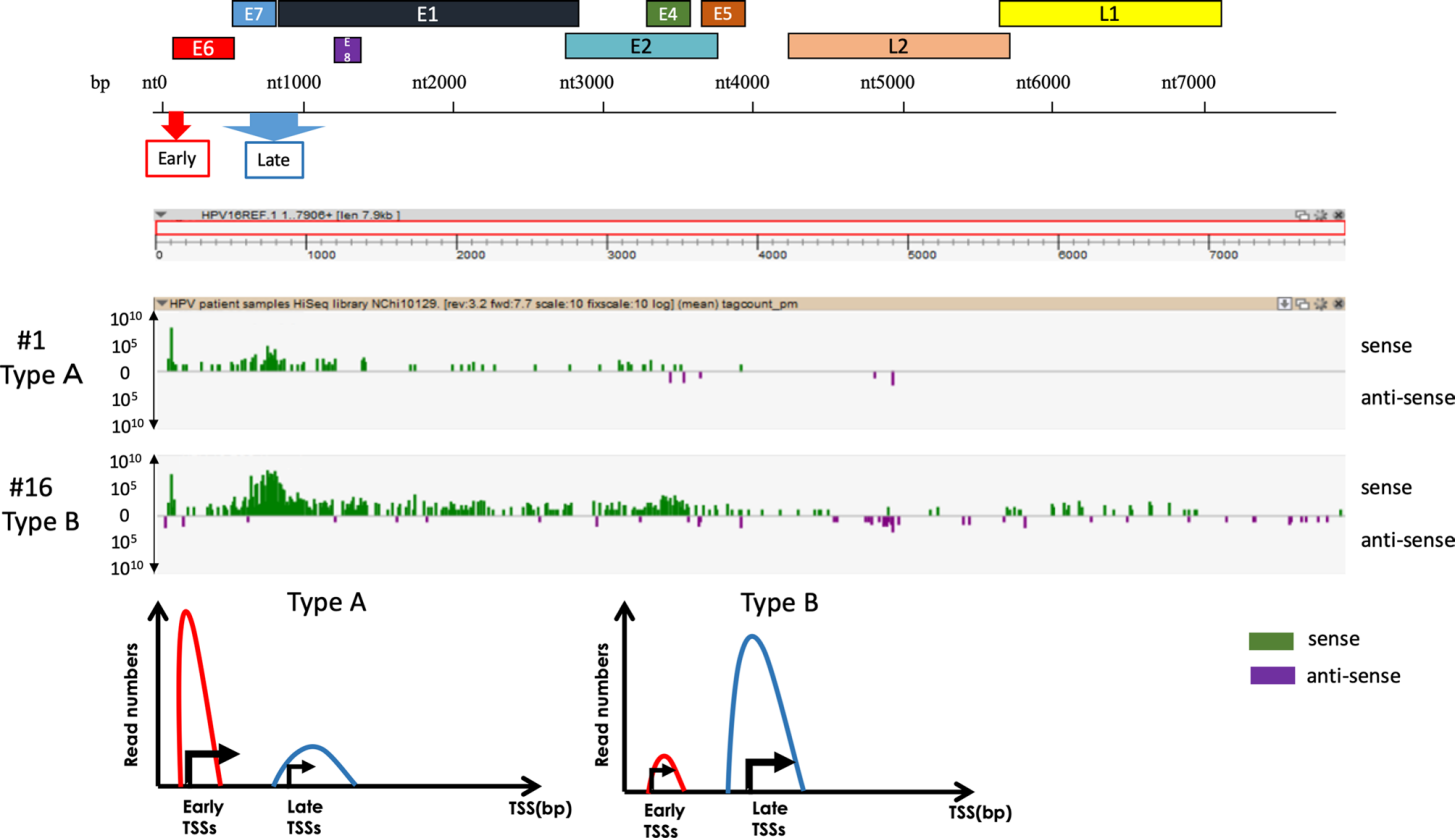
nAnTi-CAGE and nanoCAGE TSSs of HPV-positive cervical lesions. Forty-six cervical lesions, representing both normal and cancerous lesions, were analysed by nAnTi-CAGE or nanoCAGE (9 for nAnTi-CAGE and 39 for nanoCAGE). The HPV TSS patterns were investigated and classified by the prominent TSS types. Regardless of the HPV type, early and late promoter activity was defined by the numbers of TSSs in each transcriptome that started either between nucleotides 80 and 110, or between nucleotides 600 and 950. The TSS patterns were defined as follows: Type A, one-third of early promoter activity ≥ late promoter activity; Type B, one-third of early promoter activity < late promoter activity. To visualise TSS levels at the single-nucleotide level, nanoCAGE data were visualised using ZENBU software. Representative data for each TSS pattern are shown. *TSS* transcription start site.

**Figure 2:**
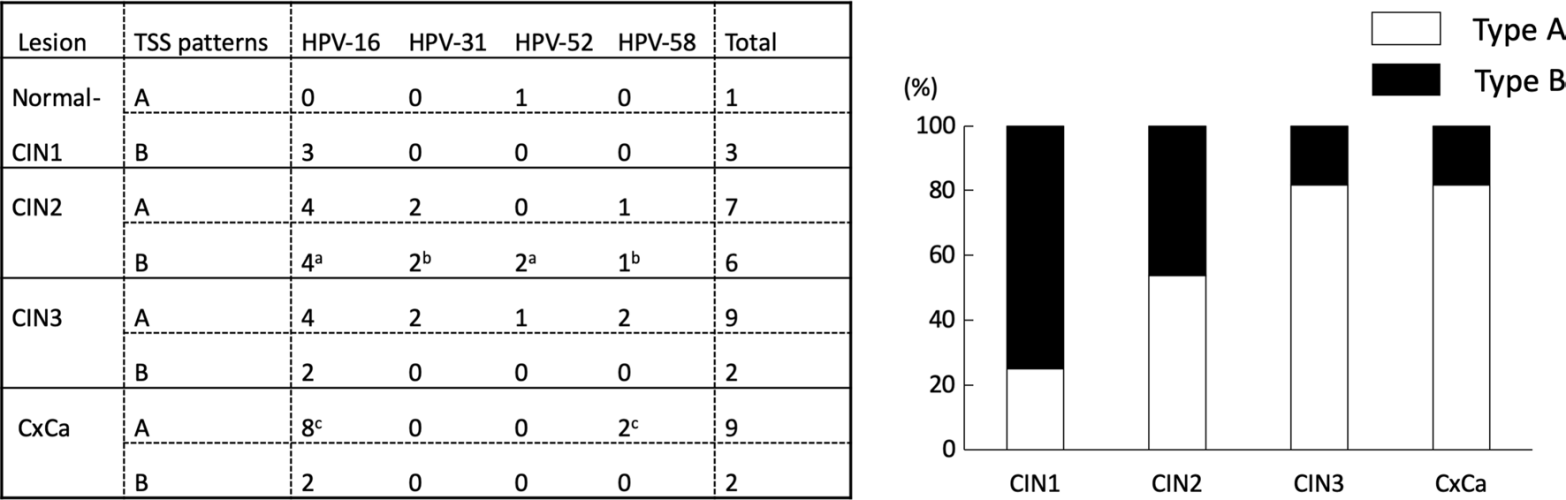
nAnTi-CAGE and nanoCAGE TSSs of HPV-positive cervical lesions. Thirty-nine cervical lesions with HPV-16, HPV-31, HPV-52, and/or HPV-58 infections, isolated from normal and cancerous lesions, were analysed by nAnTi-CAGE or nanoCAGE. The HPV TSS patterns were investigated and classified according to the prominent TSS types. Regardless of the HPV strain, early and late promoter activity was defined by the numbers of TSSs in each transcriptome that started either between nucleotides 80 and 110, or between nucleotides 600 and 950. The TSS patterns were defined as follows: Type A, one-third of early promoter activity ≥ late promoter activity; Type B, one-third of early promoter activity < late promoter activity. The TSS patterns of HPV-positive cervical lesions along with HPV type and cervical lesion status are summarised. ^a^Two samples were co-infected with HPV-16 and HPV-52. ^b^One sample was co-infected with HPV-31 and HPV-58. ^c^One sample was co-infected with HPV-16 and HPV-58. *HPV* human papillomavirus; *TSS* transcription start site; *CIN* cervical intraepithelial neoplasia; *CxCa* cervical cancer.

Forty-six cervical lesions, from normal and cancerous lesions, were analysed by nAnTi-CAGE or nanoCAGE (9 for nAnTi-CAGE and 37 for nanoCAGE). As the principle of both nAnTi-CAGE and nanoCAGE is highly similar, we first performed nAnTi-CAGE analysis for 9 samples, and we used 37 samples for nanoCAGE analysis, which is a novel technology developed after nAnTi-CAGE to meaningfully observe TSS pattern dynamics with CAGE analysis. The HPV TSS patterns were classified into broad TSS types. First, we visualised TSS activity at a single-nucleotide level using ZENBU software^26^. We identified two TSS patterns when focusing on the most activated TSS clusters: the early promoter-activated pattern and the late promoter-activated pattern, which were designated Type A and Type B, respectively. To analyse multiple HPV subtypes in parallel, we defined broad windows containing the early and late promoters in any HPV genome: from nucleotide 80 to 110, and from nucleotide 600 to 950, respectively. We discovered TSS patterns indicative of the early and late promoters, and we subsequently refined the TSS pattern definitions so that Type A included samples where one-third of early promoter activity ≥ late promoter activity; Type B, one-third of early promoter activity < late promoter activity (Fig. 1). The cervical lesion grades and corresponding HPV TSS types are summarised in Fig. 2 and Table 1. Type B was more common in CIN2 or CIN1 than in other samples, while CIN3 or cancerous lesions were predominantly Type A (chi-square test, p = 0.0224), and the observed frequency of Type B decreased with CIN progression (Cochran–Armitage test, p = 0.0208).

**Table 1:** Summary of clinical information for patients with cervical lesions.

Sample ID	Age (yr)	Stage	1st HPV	2nd HPV	TSS pattern	Early	Late	Early	Late
#34	37	NILM	52		Type A	1048	48	–	–
#40	49	NILM	–		–	–	–	–	–
#25	28	NILM	16		Type B	2	3	–	–
#13	29	CIN1	16		Type B	2	3	–	–
C1072_ATG	37	CIN1	16		Type B	441	17,349	–	–
#4	36	CIN1-2	31		Type A	71	1	–	–
#30	43	CIN1-2	52	16	Type B	1497	1355	0	6
#6	32	CIN2	31	58	Type B	1299	1422	33	41
#7	36	CIN2	31		Type A	28	5	–	–
#8	37	CIN2	58		Type A	987	130	–	–
#9	34	CIN2	16		Type A	200	9	–	–
#16	27	CIN2	16		Type B	635	7107	–	–
#23	40	CIN2	16		Type A	314	18	–	–
#24	32	CIN2	16		Type B	157	303	–	–
#26	36	CIN2	–		–	–	–	–	–
#29	38	CIN2	31		Type B	895	352	–	–
#33	39	CIN2	16		Type A	204	23	–	–
#41	45	CIN2	16		Type A	879	37	–	–
C1072_ACG	43	CIN1-2	52	16	Type B	439	12,827	9	75
#20	42	CIN2-3	–		–	–	–	–	–
#37	44	CIN2-3	16		Type B	417	1447	–	–
#1	36	CIN3	16		Type A	832	124	–	–
#3	30	CIN3	31		Type A	17	0	–	–
#12	66	CIN3	67	58	Type A	558	25	54	0
#17	47	CIN3	–		–	–	–	–	–
#18	43	CIN3	52		Type A	614	42	–	–
#19	42	CIN3	58		Type A	533	38	–	–
#21	35	CIN3	31		Type A	80	7	–	–
#22	42	CIN3	16		Type A	310	27	–	–
#39	78	CIN3	16		Type A	1932	167	–	–
#42	61	CIN3	16		Type A	238	31	–	–
C1072_GCT	30	CIN3	16		Type B	218	269	–	–
#2	31	AIS	18		Type B	7	26	–	–
#27	28	CxCa	16	58	Type A	6	2	5	0
#11	74	CxCa	16		Type A	643	99	–	–
#5	33	CxCa	16		Type A	277	40	–	–
#32	37	CxCa	18		Type B	77	58	–	–
#14	62	CxCa	58		Type A	6684	198	–	–
#38	32	CxCa	16		Type B	288	112	–	–
#43	55	CxCa	–		–	–	–	–	–
C1059_ACC	63	CxCa	16		Type A	635	114	–	–
C1059_ATG	44	CxCa	16		Type A	4939	126	–	–
C1059_ACG	59	CxCa	16		Type B	3384	2224	–	–
C1065_CAC	33	CxCa	16		Type A	3253	117	–	–
C1065_GCG	35	CxCa	16		Type A	1855	152	–	–
C1065_ATG	67	CxCa	16		Type A	1097	18	–	–

Forty-six cervical lesions, from normal and cancerous lesions, were analyzed by nAnTi-CAGE or nanoCAGE (9 for nAnTi-CAGE and 37 for nanoCAGE). The HPV TSS patterns were classified into broad TSS types: the early promoter-activated pattern and the late promoter-activated pattern, which were designated Type A and Type B, respectively. We defined broad windows containing the early and late promoters in any HPV genome: from nucleotide 80 to 110, and from nucleotide 600 to 950, respectively. TSS patterns were defined as follows: Type A, one-third of early promoter activity ≥ late promoter activity: and Type B, one-third of early promoter activity < late promoter activity.

*AIS* adenocarcinoma in situ; *CIN* cervical intraepithelial neoplasia; *CxCa* cervical cancer; *NILM* negative for intraepithelial lesion malignancy; *TSS* transcription start site.

**Figure 3:**
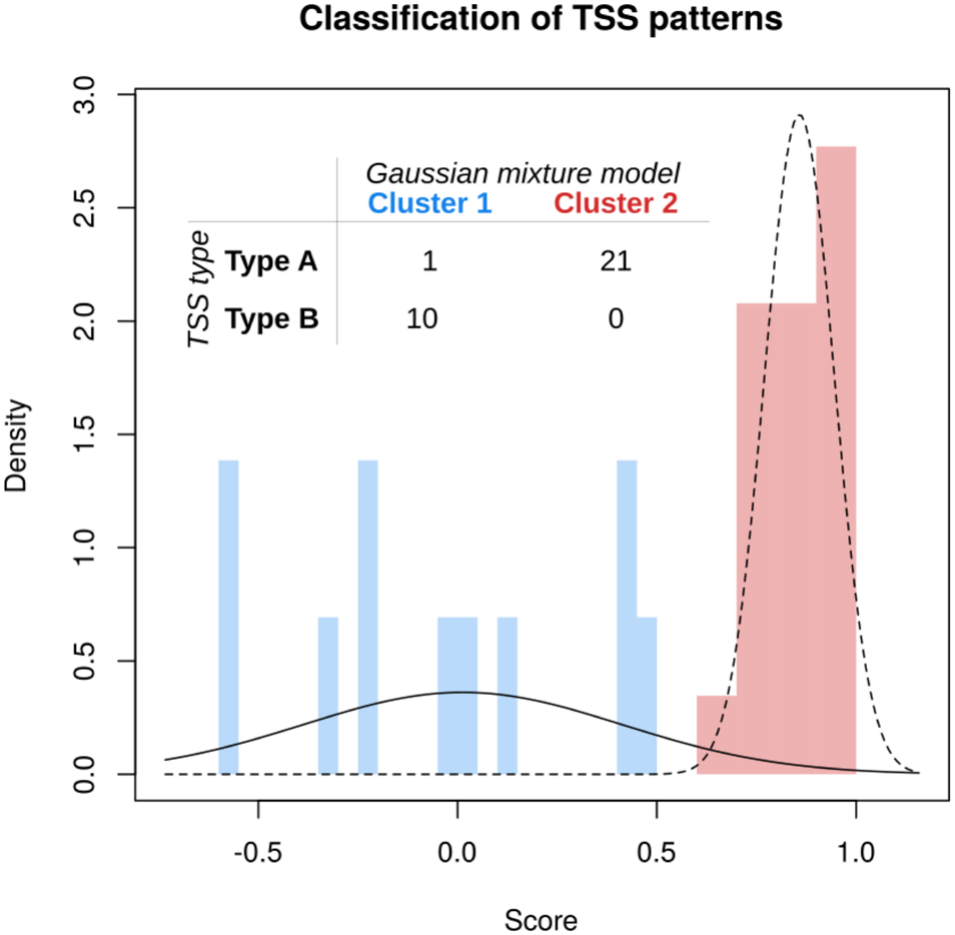
Classification of TSS patterns by Gaussian mixture models. The score was defined as the average difference between the expression levels of the early and late promoters, and a Gaussian mixture model was fitted to these scores. The model with the highest likelihood was univariate, with two components and unequal variance. The TSS patterns were classified according to the averaged difference in expression between the early and late promoters, defined as (early − late)/(early + late). *TSS* transcription start site.

We then investigated whether the initial observation of multiple TSS patterns would be supported by a more systematic approach. We fitted Gaussian mixture models^27^ to investigate the accuracy of the classified HPV-derived TSS types. Among 37 samples analyzed by nano-CAGE, 33 samples of which HPV-derived TSS was detected were included in this study. Of them, 2 samples were co-infected with two HPV genotypes. Thirty-five HPV-derived TSS types were classified by Gaussian mixture models, and compared to the types of HPV-derived TSSs classified according to the averaged difference in expression between the early and late promoters, defined as (early – late)/(early + late). The model with the highest likelihood was univariate, with two components and unequal variance: this corresponded closely to Type A and the union of Type B, since only one Type A sample (sample #27) was classified as Type B (Fig. 3).

#### TSS patterns in multiple infections

**Figure 4:**
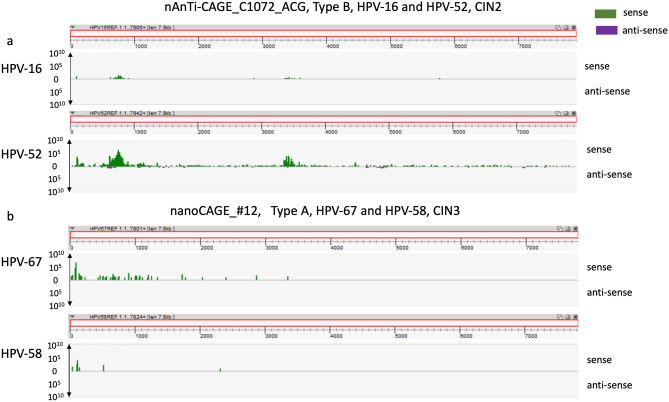
TSS patterns of cervical lesions with HPV co-infection. (**a**) nAnTi-CAGE analysis of a CIN sample co-infected with HPV-16 and HPV-52. To visualise TSS activity at a single-nucleotide level, nAnTi-CAGE data were visualised using ZENBU software. (**b**) nanoCAGE analysis of a CIN sample co-infected with HPV-67 and HPV-58. To visualise TSS activity at a single-nucleotide level, nanoCAGE data were visualised using ZENBU software. *HPV* human papillomavirus; *TSS* transcription start site; *CIN* cervical intraepithelial neoplasia.

We detected transcriptomes of multiple HPV types in five samples in the current study (Table 1). The following co-infections were observed: HPV-16 and HPV-52 (samples C1072_ACG and #30); HPV-31 and HPV-58 (sample #6); HPV-16 and HPV-58 (sample #27); and HPV-67 and HPV-58 (sample #12). Interestingly, the TSS patterns of both detected HPV types were the same in each sample (Fig. 4). Furthermore, a dominant HPV type was apparent in each case of co-infection (Fig. 4 and Table 1).

### Assessment of small promoters by nAnTi-CAGE technology

**Figure 5:**
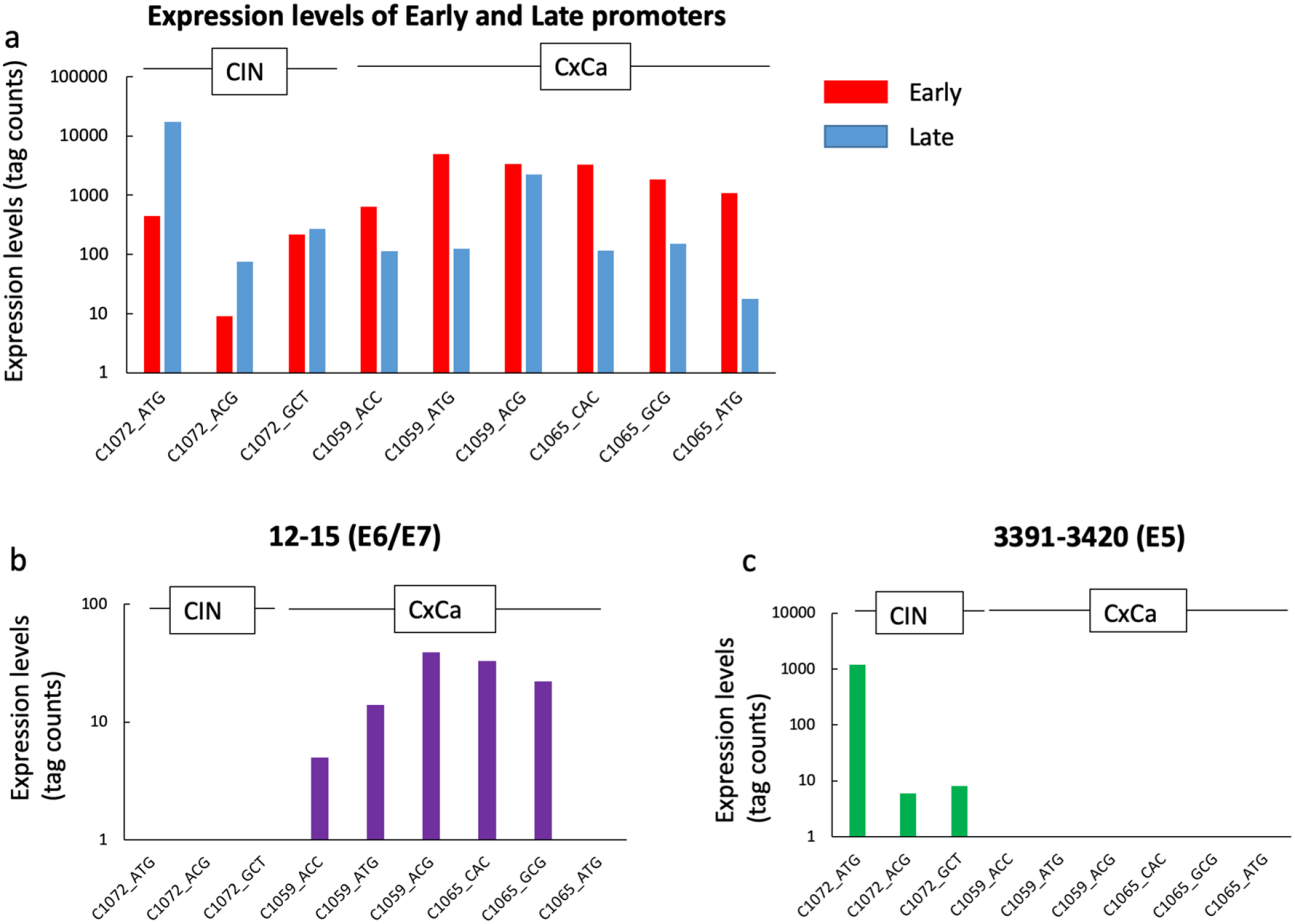
HPV-16–derived tag numbers of cervical samples by nAnTi-CAGE. (**a**) Expression levels of early and late promoters across the cervical samples. (**b**) The cluster located at nt12-15 for E6/E7 genes. (**c**) The cluster located at nt3391-3420 for E5 gene. *HPV* human papillomavirus; *TSS* transcription start site; *CIN* cervical intraepithelial neoplasia; *CxCa* cervical cancer.

In a previous study, we identified numerous HPV-derived TSS clusters in a CIN cell line and a CIN sample^13^. In the present study, we used nAnTi-CAGE to detect small HPV16-derived TSS clusters, as well as the prominent early and late promoters, in clinical samples (Table 2). One of the small TSS clusters was found to be for the E8^E2 gene, and is located at nt1125-1148. We identified the E8^E2 TSS in 3 of 6 cancer samples and 2 of 3 CIN samples. Another small TSS cluster found to be for the E5 gene, located at nt3391-3420^7^, was identified in all CIN samples. Furthermore, we also identified a cluster located at nt12-15 in all cervical cancer samples. Focusing on the early and late promoters, as well as the cluster located at nt12-15 for the E6/E7 genes and nt3391-3420 for the E5 gene, there are changes in gene expression according to the usage of each viral promoter (Fig. 5).

**Table 2:** Summary of the HPV-16-derived tag numbers of cervical samples by nAnTi-CAGE.

Start of cluster (HPV-16 nt)	End of cluster (HPV-16 nt)	Strand	C1072_ATG	C1072_ACG	C1072_GCT	C1059_ACC	C1059_ATG	C1059_ACG	C1065_CAC	C1065_GCG	C1065_ATG
**12**	**15**	**+**	**·**	**·**	**·**	**5**	**14**	**39**	**33**	**22**	**·**
90	97	+	425	9	214	616	4896	3315	3177	1801	1085
670	672	+	230	**·**	**·**	6	**·**	30	**·**	11	**·**
710	713	+	536	**·**	**·**	**·**	**·**	47	**·**	**·**	**·**
714	717	+	425	**·**	**·**	**·**	**·**	45	**·**	**·**	**·**
741	798	+	12,965	58	11	39	32	1217	26	50	6
930	932	+	16	**·**	**·**	**·**	5	**·**	**·**	**·**	**·**
952	955	+	128	**·**	**·**	**·**	17	**·**	**·**	**·**	**·**
972	974	+	6	**·**	**·**	**·**	**·**	**·**	**·**	**·**	**·**
997	999	+	7	**·**	**·**	**·**	**·**	**·**	**·**	**·**	**·**
1120	1122	+	**·**	**·**	**·**	**·**	**·**	8	**·**	**·**	**·**
**1125**	**1148**	**+**	**29**	**·**	**6**	**29**	**·**	**116**	**·**	**8**	**·**
1225	1228	+	**·**	**·**	**·**	**·**	**·**	7	**·**	**·**	**·**
1234	1236	+	**·**	**·**	**·**	**·**	**·**	**·**	**·**	**·**	**·**
1259	1262	+	20	**·**	**·**	**·**	**·**	**·**	**·**	**·**	**·**
1398	1400	+	**·**	**·**	**·**	**·**	**·**	**·**	**·**	**·**	**·**
1509	1512	+	**·**	**·**	**·**	**·**	43	**·**	**·**	**·**	**·**
1563	1566	+	**·**	**·**	**·**	**·**	5	**·**	**·**	**·**	**·**
1570	1573	+	**·**	**·**	**·**	**·**	5	**·**	**·**	**·**	**·**
1675	1678	+	11	**·**	**·**	**·**	17	**·**	**·**	**·**	**·**
2009	2011	+	**·**	**·**	**·**	**·**	**·**	**·**	**·**	**·**	**·**
2057	2060	+	**·**	**·**	**·**	**·**	**·**	**·**	**·**	**·**	**·**
3357	3361	+	25	**·**	**·**	**·**	**·**	**·**	**·**	**·**	**·**
**3391**	**3420**	**+**	**1197**	**6**	**8**	**·**	**·**	**·**	**·**	**·**	**·**
3441	3443	+	11	**·**	**·**	**·**	**·**	**·**	**·**	**·**	**·**
3444	3446	+	55	**·**	**·**	**·**	**·**	**·**	**·**	**·**	**·**
3495	3497	+	64	**·**	**·**	**·**	**·**	**·**	**·**	**·**	**·**
3585	3589	+	162	**·**	**·**	**·**	**·**	**·**	**·**	**·**	**·**
3633	3638	+	**·**	**·**	**·**	**·**	**·**	**·**	**·**	**·**	**·**
4022	4024	+	**·**	**·**	**·**	**·**	**·**	**·**	**·**	**·**	**·**
5661	5665	+	**·**	**·**	**·**	**·**	**·**	**·**	**·**	**·**	**·**
7684	7687	+	**·**	**·**	**·**	**·**	**·**	8	**·**	**·**	**·**
7782	7785	+	**·**	**·**	**·**	**·**	**·**	**·**	**·**	**·**	**·**
7852	7856	+	**·**	**·**	**·**	**·**	**·**	**·**	**·**	**·**	**·**
592	652	−	13	**·**	**·**	**·**	**·**	**·**	**·**	**·**	**·**
945	950	−	**·**	**·**	**·**	**·**	**·**	**·**	**·**	**·**	**·**
1311	1328	−	**·**	**·**	**·**	**·**	**·**	**·**	**·**	**·**	**·**
1884	1891	−	**·**	**·**	**·**	**·**	**·**	**·**	**·**	**·**	**·**
2850	2893	−	13	**·**	**·**	**·**	**·**	**·**	**·**	**·**	**·**
4852	4933	−	**·**	**·**	**·**	**·**	**·**	**·**	**·**	**·**	**·**
7561	7632	−	9	**·**	**·**	**·**	**·**	**·**	**·**	**·**	**·**

CAGE tag 5′-coordinates were used for Paraclu clustering with the following parameters: (i) minimum five tags per cluster; (ii) (maximum density/baseline density) ≥ 2; and (iii) 100-bp maximum cluster length. Tag numbers < five were designated as negative for each TSS cluster.

nt12-15, nt1125-1148, and nt3391-3420 are highlighted in bold.

“**·**” indicates negative for each TSS cluster (tag numbers < five).

## Discussion

In the present study, we noted that the TSS patterns in the HPV genome may reflect the lesion stage of infected tissue. In all cancer samples and in several CIN samples, the prominent TSS patterns corresponded to the early promoter, while in low-grade CIN samples, the dominant TSS clusters had shifted from the early to the late promoter. Furthermore, in lesions with multiple infections, the prominent TSS patterns were the same, regardless of HPV type.

Quantitative visualisation of TSS activation had previously been difficult to capture. Previous studies revealed the presence of two major promoters on the HPV genome: the early and late promoters. The activation of these promoters has been extensively investigated in reporter assays using cultured cells. However, quantitative evaluation of promoter activity in clinical samples remained challenging. Furthermore, overlapping transcripts complicated the quantitative evaluation of the individual transcripts. CAGE technology facilitates this by enabling the detection of precise TSSs and the quantitative evaluation of their activity. In the present study, we quantitatively assessed TSS activation in clinical samples using CAGE, and determined the occurrence of at least two TSS patterns in clinical samples: Type A and Type B. Considering that the expression of late genes is up-regulated in later stages of the viral life cycle, which is coordinated with epithelial differentiation, the Type B TSS pattern could represent a normal viral life cycle and correlate with lower CIN grades. As shown in Fig. 2, most of the samples with lower CIN grades (i.e. CIN1 and CIN2) showed the Type B pattern, which could represent a normal viral life cycle, while the Type A pattern accounted for a larger proportion in CIN3 and cancer samples.

Another important finding was the detection of weak TSS cluster activity, such as that of the TSS clusters at nt12-15, nt1125-1148, and nt3391-3420. In particular, in our cohort, a weak E6/E7 TSS cluster, nt12-15, was only detected in cervical cancer samples; however, an E5 TSS cluster, nt3391-3420, was only detected in CIN samples. In addition to the TSS patterns, the expression of these weak TSS clusters could serve as diagnostic biomarkers for cervical cancer progression. In the present study, we also identified the cluster at nt1125-1148, a TSS cluster of E8^E2^28^, which is regulated by E1 and E2. The E8^E2 protein plays an important role in regulating viral genome replication during the course of infection^28–31^, and E8^E2 expression inhibits the proliferation of cancer cells^32^. However, until now, there has been no evidence for the existence of E8^E2 in clinical samples. After a direct evaluation of TSSs, we report here for the first time the identification and quantitation of an activated E8 promoter in clinical samples in three of seven cervical cancers and two of three CIN samples. Further analysis and clinical follow-up of specific patients are required to elucidate the association between E8^E2 expression and cancer progression.

We then demonstrated that the TSS pattern was the same in co-infected samples, regardless of the HPV types involved. It is plausible that viral gene expression changes in parallel with the differentiation of the infected epithelial cells^33,34^. The viral gene expression pattern may thus be determined by the differentiation status of the epithelial cells, regardless of HPV type. In well-differentiated superficial cells, the HPV late promoter is activated^33,34^. In contrast, in high-grade CIN samples, lack of epithelial differentiation may be associated with a stable expression of HPV early genes, such as genes encoding the E6 and E7 oncoproteins.

We originally defined the A and B TSS patterns based on visual inspection of the data, and defined Type A as having early > late promoter activity, and Type B as having late > early promoter activity. Independent classification based on Gaussian mixture models suggested that these definitions could be refined using machine learning. Nevertheless, we demonstrated the feasibility of a novel method for the evaluation of altered HPV promoter activity during disease progression in clinical samples. This constitutes a proof-of-principle for the utility of TSS patterns as a diagnostic marker for CIN severity or progression. An extended CAGE study with more samples would allow for further assessment of the possibility of linking TSS patterns to disease state. Such a study would need to balance the requirement for screening a large number of samples with the requirement for sequencing a sufficiently high number of reads from each sample. Using the classification proposed in the present study, distinguishing between Type A and B patterns required at least 16 tags (in total) for the early and late promoters. The HPV transcriptome represents only a fraction of the available sequence libraries, varying roughly between 1 per million and 1 per cent. Therefore, either new samples should be sequenced at a depth of 10 to 20 million reads, or an enrichment method should be developed to address this issue. A limitation of the present study was that the number of clinical samples was not sufficient to allow statistical validation of the association between different TSS patterns and the severity of CIN lesions, or the differentiation status of the epithelium.

In conclusion, in this study we demonstrated the feasibility to analyse TSS activity at the single-nucleotide level using CAGE technology in clinical HPV samples. Further work on a larger cohort following the same patients over time will be needed for determining the sensitivity and specificity of the quantification of dynamic changes of TSS patterns as a biomarker of disease progression.

## Methods

### Patients and clinical samples

HPV-infected cervical tissues were obtained from biopsy or surgery samples. Diagnosis was confirmed by experienced pathologists and gynaecological oncologists through pathological and colposcopic examination at the University of Tokyo Hospital. HPV-infected cervical tissues were also examined by H&E staining, and the extent of dysplasia was evaluated. Cervical intraepithelial neoplasia was categorised as grade 1, 2, and 3 (CIN1, CIN2, and CIN3) depending upon the proportion of abnormal cell thickness. Then, experienced gynaecological oncologists confirmed the biopsied samples as CIN or a cervical cancer lesion. The samples for CAGE analysis were taken from the same area that met the diagnostic criteria of a cervical lesion. All experimental procedures were approved by the institutional review board at The University of Tokyo (approval number G0637-6), and signed informed consent for the use of the tissues and genomic data was obtained from each participant. Preparation of nanoCAGE libraries at RIKEN was approved by the institutional review board at the Yokohama Campus (approval number H26-26). For the analysis, RNA (5 µg for nAnTi-CAGE and 500 ng for nanoCAGE) was extracted from each sample using an miRNeasy kit (Qiagen, Hilden, Germany). RNA quality was assessed using a Bioanalyzer (Agilent) and standardised to an RNA integrity number (RIN) of > 7.0 for nAnTi-CAGE or > 5.0 for nanoCAGE. The purity of RNA samples was assessed using NanoDrop analysis, which confirmed that the *A*_260_/*A*_290_ and *A*_260_/*A*_230_ ratios were > 1.7.

### Ethical considerations

This study was approved by the institutional review board at The University of Tokyo (approval number G0637-6) in accordance with the Declaration of Helsinki. All patients provided written informed consent for study participation.

### nAnTi-CAGE library construction

First-strand cDNA was transcribed to include the 5′-end of capped RNA, and CAGE ‘barcode’ tags were attached as previously described^35^. The sequenced CAGE tags were mapped to the HPV-16 and HPV-52 genome based on the infected HPV genotypes using BWA software (v0.5.9), discarding ribosomal or non-A/C/G/T base-containing RNAs. For the HPV-16 genes, CAGE tag 5′-coordinates were used for Paraclu clustering^36^ with the following parameters: (i) minimum five tags per cluster; (ii) (maximum density/baseline density) ≥ 2; and (iii) 100-bp maximum cluster length. Tag numbers < 5 were designated as negative for each TSS cluster.

### nanoCAGE library construction

nanoCAGE libraries were constructed from isolated RNA as previously described^12^, with some modifications. The reverse-transcription products were eluted in 40 µL, and qPCR was conducted using the SYBR Premix Ex Taq kit (TaKaRa). Cycle numbers were estimated as Ct + 4 cycles, and PCR was conducted to generate cDNA using the Ex Taq enzyme (TaKaRa). PCR products were eluted in 30 μL of sterile distilled water after purification, and 0.3 ng of each sample was tagmented individually at 55 °C for 5 min. The extension time of the final PCR was 30 s, and the final purification was achieved using one volume of AMPure reagent (Beckman Coulter, Inc), with the products eluted in 25 μL of reaction mixture. The multiplexed libraries were then paired-end sequenced in five lanes of a HiSeq 2000 sequencer (Illumina) and aligned to human genome version hg38 supplemented with all the HPV genomes available on the Papillomavirus Episteme database^37^ on 5 Sep, 2016, using the CAGEscan pipeline v3.0 (https://gitlab.com/mcfrith/cagescan-pipeline, Kratz et al., in preparation), which assembles overlapping pairs originating from the same molecule and maps them to the genome using the LAST aligner^38^.

### Statistics

The association between cervical lesion grades and TSS types was evaluated by the chi-square test and Cochrane–Armitage test using JMP Pro version 12.2.0 (SAS Institute, Cary, NC, USA). A p value < 0.05 was considered statistically significant. The ‘densityMclust’ function in the R package mclust v5.4^27^ was used to compare the likelihood of different Gaussian mixtures. After defining a score as the average difference between the expression levels of the early and late promoters, we fitted Gaussian mixture models to these scores. The TSS patterns were classified according to the averaged difference in expression between the early and late promoters, calculated as (early—late)/(early + late). The minimum number of samples required for achieving confidence < 0.25 (i.e. to distinguish between Types A and B) was determined using the ‘ciss.wald’ function in the R package binomSamSize v0.1–5 (Fig. 3).

## Data Availability

Demultiplexed sequence files are being submitted to the Japanese Genotype–Phenotype Archive (JGA).
